# Biatrial Macroreentrant Tachycardia Involving Persistent Left Superior Vena Cava and Bachmann's Bundle

**DOI:** 10.1016/j.jaccas.2026.107049

**Published:** 2026-03-18

**Authors:** Chun-Xuan Wu, Yu-Li Yang, Xiang-Dong Liu, Xing-Xing Cai, Wei Li, Yi-Gang Li

**Affiliations:** Department of Cardiology, Xinhua Hospital Affiliated to Shanghai Jiao Tong University School of Medicine, Shanghai, China

**Keywords:** ablation, atrial fibrillation, atrial flutter, atrial tachycardia, electroanatomic mapping

## Abstract

**Background:**

Atrial tachycardia after atrial fibrillation ablation may arise from uncommon epicardial conduits.

**Case Summary:**

A 73-year-old man with persistent atrial fibrillation underwent redo ablation for recurrent atrial tachycardia. Electroanatomic mapping showed durable pulmonary vein and posterior wall isolation with a blocked anterior mitral line. Tachycardia (cycle length: 252 milliseconds) appeared mitral isthmus–dependent, but endocardial ablation only prolonged the cycle without termination. Mapping revealed a macroreentrant biatrial tachycardia incorporating the persistent left superior vena cava (PLSVC) and Bachmann's bundle (BB). Given the central role of the PLSVC, targeted ablation inside the PLSVC terminated the tachycardia and confirmed mitral isthmus block.

**Discussion:**

This rare case illustrates how both the PLSVC and BB can serve as epicardial substrates for biatrial macroreentry, emphasizing comprehensive mapping and tailored ablation in redo procedures.

**Take-Home Messages:**

Recognition of epicardial conduction via PLSVC and BB is essential in redo ablations.

## Visual Summary

**Figure:**
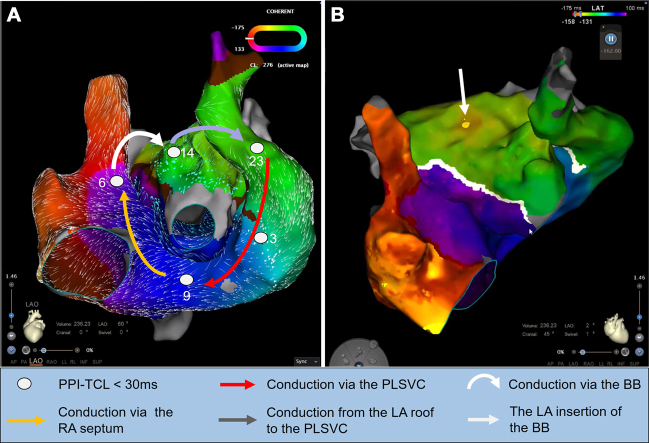

**Visual Summary:**
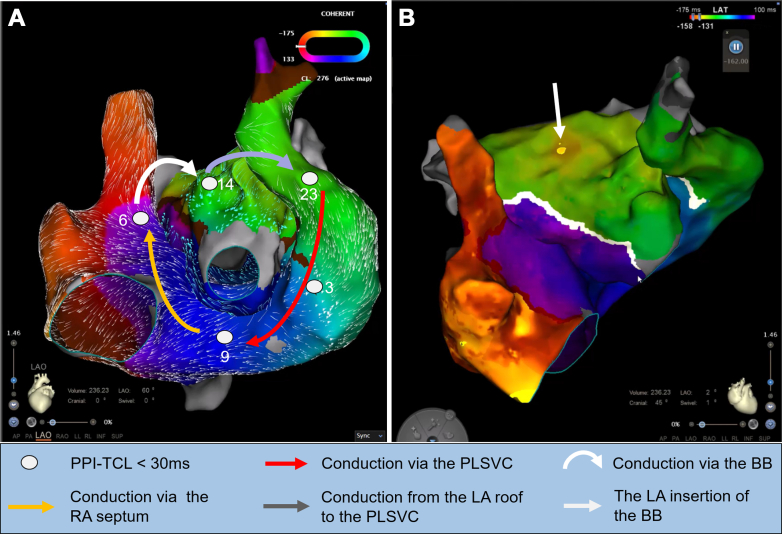
Biatrial Macroreentrant Tachycardia Involving the PLSVC and BB (A) High-resolution activation mapping demonstrates a macroreentrant biatrial tachycardia incorporating 2 epicardial pathways: the PLSVC and BB. Entrainment pacing at the PLSVC, the RA septum, and the BB insertion site showed PPI–TCL differences of <30 milliseconds, supporting their critical participation in the reentrant circuit. (B) Detailed LA roof view showing the BB insertion at the anterior roof of the LA adjacent to a blocked anterior mitral line. BB = Bachmann's bundle; LA = left atrial; PLSVC = persistent left superior vena cava; PPI-TCL = postpacing interval–tachycardia cycle length; RA = right atrial.

## History of Presentation

**Figure 1:**
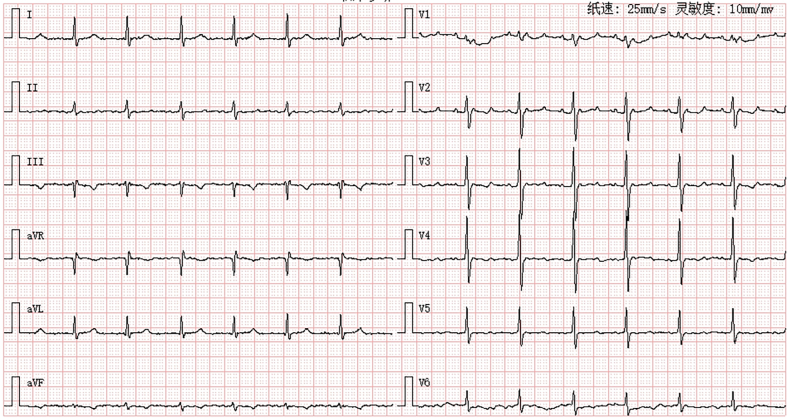
Surface ECG Demonstrating Atrial Tachycardia Twelve-lead electrocardiogram (ECG) shows organized atrial activity with a 3:1 atrioventricular conduction.

A 73-year-old man with persistent atrial fibrillation underwent redo ablation for recurrent symptomatic atrial tachycardia. The patient presented with exertional dyspnea and discomfort. Coarse breath sounds were noted bilaterally, accompanied by scattered moist rales at the lung bases. Mild bilateral lower extremity edema was observed. Laboratory testing revealed elevated N-terminal pro–B-type natriuretic peptide at 1,082 pg/mL. The surface electrocardiogram demonstrated atrial tachycardia with 3:1 atrioventricular conduction and a regular ventricular rate of 78 beats/min (Figure 1).

**Take-Home Messages**

• Epicardial structures such as the PLSVC and Bachmann's bundle can sustain macroreentrant tachycardia.
• Recognition of these conduits is crucial for accurate diagnosis and effective ablation in redo cases.

## Past Medical History

Six months earlier, the patient underwent a 1-stop procedure with radiofrequency ablation for persistent atrial fibrillation combined with left atrial appendage (LAA) occlusion. At that time, bilateral pulmonary vein isolation, roof line, posterior wall “box” isolation, mitral isthmus line, anterior line, and tricuspid isthmus line ablation were performed, and sinus rhythm was restored by cardioversion. He also had a >10-year history of hypertension.

## Differential Diagnosis

In a patient with a history of persistent atrial fibrillation and prior extensive left atrial ablation presenting with organized atrial tachycardia, several possibilities should be considered:

1. Focal atrial tachycardia. It may arise from atrial scars or residual musculature after prior ablation. A focal-appearing breakthrough on activation mapping does not inevitably represent a true focal atrial tachycardia and should be interpreted in the context of entrainment mapping to distinguish focal from reentrant mechanisms.
2. Typical atrial flutter. It could be readily evaluated by combining surface electrocardiography with activation and entrainment mapping, allowing this mechanism to be confidently excluded.
3. Atypical atrial flutter. Atypical atrial flutter was strongly considered given the patient's history of extensive prior left atrial ablation, which can create complex reentrant circuits involving ablation lines or atrial scars. Sometimes the involvement of epicardial conduction pathways should be noted.

## Investigations

**Figure 2:**
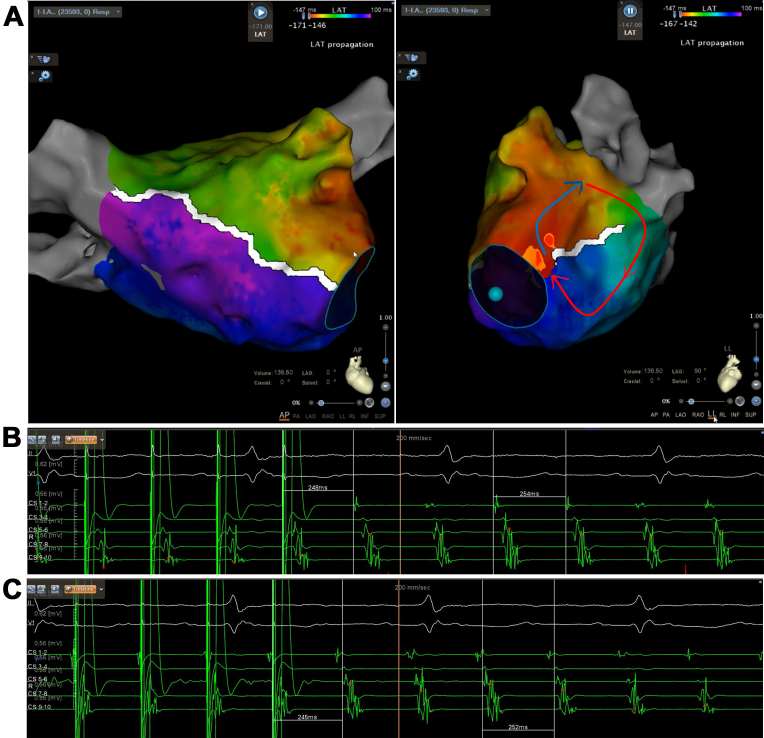
Activation and Entrainment Mapping in the Left Atrium (A) Activation mapping indicated a blocked anterior mitral line, incomplete block across the mitral isthmus line, and the tachycardia cycle length could not be fully accounted for in the left atrium. The postpacing intervals were almost equal to the tachycardia cycle length at CS 1 and CS 2 (B), and CS 5 and CS 6 (C).

**Figure 3:**
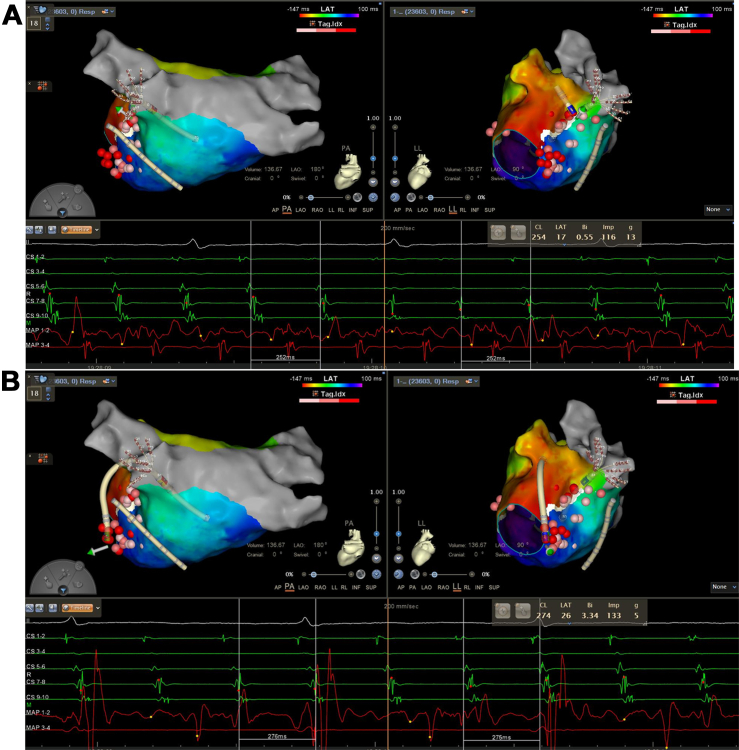
Prolongation of the Atrial Tachycardia by Ablation Along the Mitral Isthmus Line (A) Ablation at the pulmonary vein side of the mitral isthmus line did not alter the tachycardia cycle length, (B) whereas ablation at the annular side of the mitral isthmus line prolonged the tachycardia cycle length from 252 to 275 milliseconds.

High-density electroanatomic mapping confirmed durable isolation of all pulmonary veins, a successfully electrically isolated left atrial posterior wall, and a blocked anterior mitral line. The tachycardia cycle length was 252 milliseconds. Although a full endocardial reentry loop was not mapped during activation mapping, entrainment pacing from coronary sinus (CS) 1 to 2 and CS 5 to 6 demonstrated postpacing intervals closely matching the tachycardia cycle length, supporting a mitral isthmus–dependent reentry and suggesting involvement of the epicardial pathway (Figure 2). Subsequent endocardial ablation along the isthmus only prolonged the cycle to 275 milliseconds without termination, and coronary sinus activation was unchanged (Figure 3).

Ethanol ablation in the vein of Marshall was considered. However, coronary venography revealed the presence of persistent left superior vena cava (PLSVC). High-resolution biatrial mapping uncovered a rare macroreentrant biatrial tachycardia involving 2 epicardial pathways: clockwise activation propagated from the PLSVC to the right atrium, ascended along the right atrial septum to the superior vena cava, and crossed into the left atrium (LA) via the Bachmann's bundle (BB) (Video 1). The breakthrough site of the BB at the left atrial anterior roof was clearly shown. It then traveled along the left atrial roof to the LAA, and reentered the PLSVC via epicardial connections between the LAA and PLSVC, completing the loop (Video 1). The postpacing intervals were almost equal to the tachycardia cycle length at the right atrial septum, left atrial roof, and the PLSVC.

## Management

**Figure 4:**
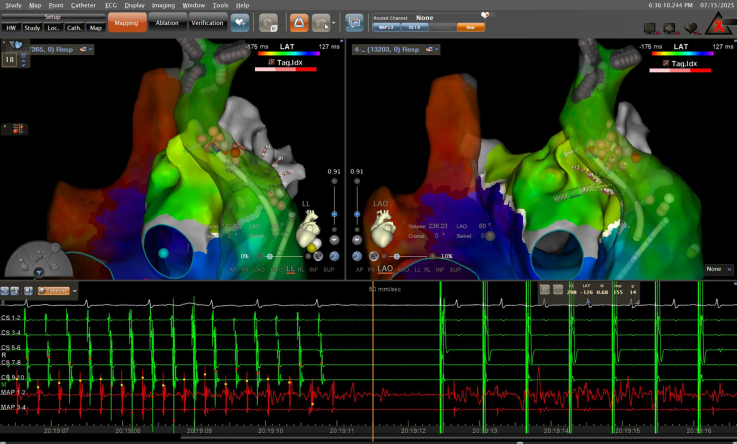
Termination of the Atrial Tachycardia by Ablation Within the PLSVC Radiofrequency ablation targeting the connection of the left atrium and persistent left superior vena cava (PLSVC) resulted in immediate termination of the atrial tachycardia. Sinus arrest occurred, and temporary pacing was immediately initiated for cardiac protection.

**Figure 5:**
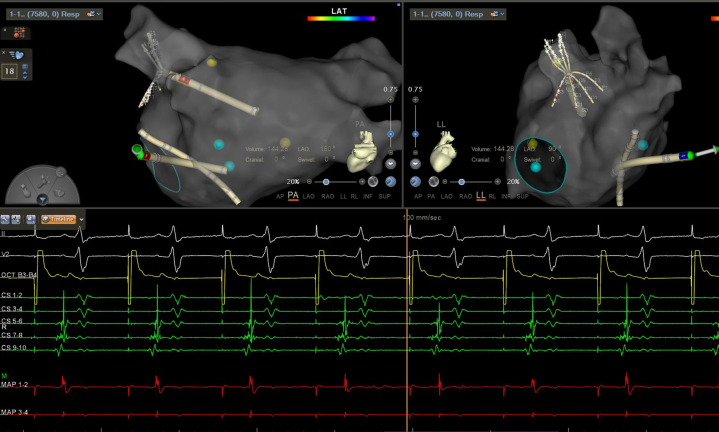
Confirmation of Mitral Isthmus Block by LAA Pacing Pacing from the left atrial appendage (LAA) produced a proximal-to-distal activation sequence in the coronary sinus, indicating conduction block across the mitral isthmus line.

For the biatrial tachycardia involving the PLSVC and BB, 3 potential ablation targets were considered: (1) the PLSVC; (2) the left atrial insertion of the BB; and (3) its superior vena cava insertion. Considering the central role and arrhythmogenic substrate within the PLSVC, targeted ablation within the PLSVC was performed, resulting in immediate arrhythmia termination (Figure 4). LAA pacing confirmed mitral isthmus block (Figure 5).

## Outcome and Follow-Up

After the procedure, the patient developed marked sinus bradycardia with episodes of sinus arrest, for which a dual-chamber pacemaker was implanted. During 3 months of follow-up, no recurrence of atrial arrhythmia was observed, and the patient’s symptoms of heart failure markedly improved.

## Discussion

Epicardial conduction structures such as the PLSVC and BB are increasingly recognized as important contributors to atrial arrhythmogenesis. Their role becomes particularly relevant in patients undergoing repeat ablation, where conventional endocardial conduction pathways have been disrupted.

## Electrophysiological Role of the PLSVC

The PLSVC is a congenital venous remnant with well-developed myocardial sleeves that can generate ectopic activity or participate in reentrant circuits. It has been reported as a source of atrial fibrillation triggers and as a substrate for atrial tachycardia. Its muscular connections to the LA and coronary sinus provide potential epicardial conduction routes that can sustain macroreentry, especially after prior pulmonary vein isolation. Ablation within the PLSVC or ethanol infusion into the vein of Marshall has been used to eliminate such epicardial substrates.^1^

## Electrophysiological Role of the BB

The BB is the principal epicardial interatrial connection, ensuring rapid conduction from the right atrium to the LA under physiological conditions. Structural remodeling, scarring, or conduction delay within the BB region has been implicated in interatrial conduction disturbances and may predispose to atrial fibrillation. In the setting of atrial tachycardia, intact conduction through the BB can act as a critical epicardial bridge sustaining macroreentrant circuits, particularly when other interatrial pathways are disrupted by ablation.

## Mechanistic Insights From the Present Case

In our patient, both the PLSVC and the BB simultaneously participated in a macroreentrant biatrial tachycardia. The preexisting anterior mitral line block likely redirected interatrial conduction preferentially toward the BB, thereby incorporating it into the reentry. The PLSVC served as a central epicardial substrate, whereas the BB provided the interatrial link necessary to complete the circuit. This dual epicardial involvement is exceedingly rare but underscores the complexity of postablation arrhythmias.

The PLSVC may also participate in the first tachycardias (Figure 2). However, we did not map in the PLSVC to demonstrate its involvement. Based on the available data, a possible reentrant pathway of the first mapping was inferred: activation propagated from the PLSVC to the annular side of the mitral isthmus line, crossed a conduction gap on the annular aspect, ascended to the superior portion of the mitral isthmus line, and then traveled toward the connection between the LAA and the PLSVC, reentering the PLSVC to initiate the next cycle (Figure 2). Although the PLSVC might participate in both the first and second tachycardias, the reentrant pathways were fundamentally different. After ablation of the annular conduction gap, this circuit was interrupted, and the tachycardia converted to a second atrial tachycardia, which was subsequently confirmed to be a biatrial macroreentrant tachycardia.

The development of significant sinus bradycardia and sinus arrest after ablation within the PLSVC may have been multifactorial. First, long-standing persistent atrial fibrillation itself could have contributed to intrinsic sinus node dysfunction. Second, possible injury to the autonomic ganglionated plexi surrounding the PLSVC. The PLSVC and the adjacent coronary sinus region are known to harbor rich parasympathetic and sympathetic ganglionated plexi, which modulate sinus node automaticity and atrioventricular conduction. Radiofrequency energy delivery within the PLSVC could have caused partial denervation or transient autonomic imbalance, resulting in marked sinus node suppression.^2^

## Clinical Implications

This case highlights several important clinical points. First, epicardial structures should be suspected when recurrent atrial tachyarrhythmias persist despite extensive endocardial ablation. Second, high-density mapping and entrainment maneuvers are indispensable to delineate concealed epicardial conduction. Finally, ablation strategies must be tailored to target critical epicardial pathways such as the PLSVC or BB when they form essential components of the reentrant circuit. Recognizing the interplay between epicardial structures broadens therapeutic options and may improve procedural success in complex redo ablations.

## Conclusions

Epicardial structures, specifically the PLSVC and BB, can form critical components of biatrial tachycardia circuits. Detailed mapping and selective ablation are key to successful management in complex redo procedures.

## Funding Support and Author Disclosures

This work was supported by the National Natural Science Foundation of China (grant number 82570370). The authors have reported that they have no relationships relevant to the contents of this paper to disclose.

## Appendix

Video 1 High-Density Mapping of Demonstrating Biatrial Tachycardia Through Both the Persistent Left Superior Vena Cava and the Bachmann's Bundle The video shows the isolation of all pulmonary veins, a successfully electrically isolated left atrial posterior wall (BOX isolation), and a blocked anterior mitral line. The breakthrough site of the Bachmann bundle's at the anterior roof of the left atrium is clearly shown.
